# Long-Lasting Immunity Against SARS-CoV-2: Dream or Reality?

**DOI:** 10.3389/fmed.2021.770381

**Published:** 2021-11-25

**Authors:** Daniel Gussarow, Agnes Bonifacius, Anne Cossmann, Metodi V. Stankov, Philip Mausberg, Sabine Tischer-Zimmermann, Nina Gödecke, Ulrich Kalinke, Georg M. N. Behrens, Rainer Blasczyk, Britta Eiz-Vesper

**Affiliations:** ^1^Institute of Transfusion Medicine and Transplant Engineering, Hannover Medical School, Hanover, Germany; ^2^Department of Rheumatology and Clinical Immunology, Hannover Medical School, Hanover, Germany; ^3^Institute for Experimental Infection Research, TWINCORE, Centre for Experimental and Clinical Infection Research, A Joint Venture Between the Helmholtz Centre for Infection Research and Hannover Medical School, Hanover, Germany; ^4^Cluster of Excellence - Resolving Infection Susceptibility (RESIST, EXC 2155), Hannover Medical School, Hanover, Germany; ^5^Institute for Experimental Infection Research, TWINCORE, Centre for Experimental and Clinical Infection Research, A Joint Venture Between the Helmholtz Centre for Infection Research and the Hannover Medical School, Hanover, Germany

**Keywords:** antiviral T cells, immune protection, SARS-CoV-2, cellular immunity, humoral immunity

## Abstract

Since its declaration as a pandemic in March 2020, SARS-CoV-2 has infected more than 217 million people worldwide and despite mild disease in the majority of the cases, more than 4.5 million cases of COVID-19-associated death have been reported as of September 2021. The question whether recovery from COVID-19 results in prevention of reinfection can be answered with a “no” since cases of reinfections have been reported. The more important question is whether during SARS-CoV-2 infection, a protective immunity is built and maintained afterwards in a way which protects from possibly severe courses of disease in case of a reinfection. A similar question arises with respect to vaccination: as of September 2021, globally, more than 5.2 billion doses of vaccines have been administered. Therefore, it is of utmost importance to study the cellular and humoral immunity toward SARS-CoV-2 in a longitudinal manner. In this study, reconvalescent COVID-19 patients have been followed up for more than 1 year after SARS-CoV-2 infection to characterize in detail the long-term humoral as well as cellular immunity. Both SARS-CoV-2-specific T cells and antibodies could be detected for a period of more than 1 year after infection, indicating that the immune protection established during initial infection is maintained and might possibly protect from severe disease in case of reinfection or infection with novel emerging variants. Moreover, these data demonstrate the opportunity for immunotherapy of hospitalized COVID-19 patients via adoptive transfer of functional antiviral T cells isolated from reconvalescent individuals.

## Introduction

Since the outbreak of coronavirus disease 2019 (COVID-19) and its development into a pandemic in March 2020 (1), it is becoming increasingly important to understand the role of the human immune system during and after an infection with severe acute respiratory syndrome coronavirus 2 (SARS-CoV-2) in order to establish suitable measures against this virus. One major outcome of the evolving understanding has been the development of vaccines, which successfully elicit a functional immune response (2, 3). For the most part, the SARS-CoV-2 Spike (S) protein was chosen as the sole immunogenic agent, which might result in a different composition of the individual immunological memory compared to that of a natural infection (4). In any case, a better understanding of the immunological memory, especially for SARS-CoV-2, is crucial for guiding public health actions as well as improving vaccines (5). Data from SARS and Middle East Respiratory Syndrome (MERS) epidemics suggest that cellular immunity to these viruses can last for many years (6, 7), but for SARS-CoV-2, this has yet to be investigated. Recent studies detected the presence of cellular immunity as well as humoral immunity for up to twelve months post infection (8–12). Nevertheless, it is highly challenging to determine reliable correlates of sufficient immunological protection against reinfection, severe disease, or other outcomes (13).

One initial question to be answered is the probability of reinfections, which have already been reported in recent studies (14, 15). Apparently, the rate of reinfection or infection in vaccinated subjects is increasing (14). However, based on current knowledge, these cases are generally milder in symptoms and only contribute to a small percentage of infections (16). Nevertheless, emerging virus variants can continuously acquire enhanced transmissibility as well as higher immune-escape potential (17, 18). It is therefore of great interest to investigate and understand the immune response toward those variants and their effects on its functionality.

Such variants of concern (VOCs) have repeatedly emerged and progressed rapidly from causing only a small percentage of infections to being the dominant circulating virus variant (19). During the current pandemic, processes of that kind have been observed with alpha (B.1.1.7), beta (B.1.351), and delta variants (B.1.617.2) (19–21). Consequentially, this has to be taken into account for future decisions in public health policies and therapeutic developments.

The aim of this study was to provide follow-up data on a group of mostly non-hospitalized convalescent COVID-19 patients over a period of more than 1 year after SARS-CoV-2 infection. Distinctly, this study presents cellular and humoral immunity data of individual convalescent COVID-19 patients in order to investigate both pillars of the specific immune response over the observed time period. We examined cellular immune profiles as well as corresponding frequencies of specific T cells against SARS-CoV-2, endemic coronavirus strains (huCoV) OC43 and 229E, human Respiratory Syncytial Virus (RSV), H1N1 Influenza A Virus (IAV), and Cytomegalovirus (CMV). Furthermore, SARS-CoV-2 antibody levels were measured in order to assess humoral immunity. By analyzing the data of the whole cohort as well as for individual long-term follow-ups, we present a broad picture of SARS-CoV-2 related immunological memory along with individual progressions in this study. Additionally, we provide data for T cell cross-functionality in regards to alpha and beta virus variants. SARS-CoV-2-specific T cell responses and S-specific antibody levels remained stable after an initial decrease during the first 6–12 months of convalescence. In one case of reinfection, a rapid increase of the humoral immune response was observed. Together, these data indicate that the adaptive immunity developed during SARS-CoV-2, albeit on a low level, remains stable and possibly protective on the long-term.

## Materials and Methods

### Study Population

This study was approved by the Internal Review Board of Hannover Medical School (MHH, approval number 3639_2017, 9001_BO-K) and includes 206 donors, who gave written informed consent for their peripheral blood to be collected and used for research purposes at Hannover Medical School (MHH). All donors are recovered COVID-19 patients, who were sampled between 14 and 433 days after symptom onset. This study population consists of 98 female and 108 male donors, being 18 to 69 (Median = 46.25) years old (Table 1). For PBMC isolation, either whole blood samples or residual blood samples from platelet and plasma disposables were used. From each donor, two to thirteen samples were collected between April 2020 and May 2021.

**Table 1:** Description of the COVID-19 recovered donor cohort.

	**Donor cohort**				**Individual** **follow-up Cohort**
	**Total**	**0–26 weeks**	**27–52 weeks**	**53+ weeks**	
Gender (n, female/male)	98/108	95/98	27/55	10/14	9/9
Age (median, range)	46.4 (18–69)	45.6 (18–68)	48.5 (19–69)	49.8 (25–69)	49.2 (24–68)
WHO clinical progression scale (median, range)	2 (1–7)	2 (1–7)	2 (1–5)	2 (2–4)	2 (2–4)

*Shown are absolute numbers (gender distribution) or median and range (age and WHO clinical progression scale)*.

### Clinical Definition of Cohort

All donors had been infected with SARS-CoV-2, confirmed by reverse transcriptase polymerase chain reaction (RT-PCR). The dates of symptom onset (ranging from February 2020 to October 2020) and offset of symptoms (ranging from March 2020 to November 2020) were documented. Consequentially, symptomatic periods ranged from zero (“symptom-free”) to 73 days (Median = 12 days). Disease severity was categorized by the WHO clinical progression scale (22). In this study population, the WHO score ranged from one (ambulatory) to seven (severe disease with mechanical ventilation required).

### Serological Testing by ELISA

Serology for SARS-CoV-2 was performed by ELISA (anti-SARS-CoV-2 S1 spike protein domain IgG, QuantiVac, #EI 2606-9601-10 G and anti-SARS-CoV-2 NCP IgG; Euroimmun, Lübeck, Germany, #EI 2606-9601-2 G) according to the manufacturer's instructions (plasma dilution 1:500). Antibody levels are expressed as IgG RU/ml (RU= Relative units) or IgG Ratio (optical density divided by calibrator < 0.8, negative; 0.8–1.1, intermediate; >1.1, positive).

### Cellular Immune Profiling by Flow Cytometry

Flow cytometry was performed on the FACSCanto 10c system (BD Biosciences, Heidelberg, Germany) using the BD FACSDiva Software version 8.0.1. The following markers for innate leukocytes as well as T and B cells were applied to determine the cellular immune status (Panel 1) and B cell phenotype (Panel 2): anti-CD3 fluorescein isothiocyanate (FITC, BD Biosciences, #345764), anti-CD4 Peridinin-Chlorophyll-Protein (PerCP, BD Biosciences, #345770), anti-CD8 allophycocyanin (APC, BD Biosciences, #345775), anti-CD14 Brilliant Violet 510 (BV510, BD Biosciences, #563079), anti-CD19 AF-700 (BD Biosciences, #557921) or BV510 (BD Biosciences, #302242), anti-CD20 APC-cyanine 7 (APC-Cy7, BioLegend, #302314), anti-CD24 PerCP (BioLegend, #311114), anti-CD27 BV421 (BioLegend, 356418), anti-CD38 APC (BioLegend, #356606), anti-CD45 APC-H7 (BD Biosciences, #641417) or AlexaFluor 700 (AF-700, BioLegend, #368514), anti-CD56 phycoerythrin (PE, BD Biosciences, #345812), anti-CD45RA BV605 (BioLegend, #304134), anti-CD62L BV421 (BioLegend, #304828) or BV510 (BioLegend, #304844), anti-IgM FITC (BioLegend, #314506), and anti-IgD PE (BioLegend, #348204). Cells were stained at room temperature and in the dark for 30 minutes either before (Panel 1) or after (Panel 2) lysis of erythrocytes according to the manufacturer's instructions (Panel 1: BD Biosciences, #349202, Panel 2: Beckman Coulter, Brea, CA, USA, #A07799). For the analysis of total cell numbers, whole blood samples were measured on a single platform involving TruCount™ tubes (BD Biosciences, #340334).

### Detection of Antiviral T Cell Frequencies by IFN-γ Enzyme-Linked Immunospot Assay

SARS-CoV-2-specific T lymphocytes were detected by Interferon-gamma (IFN-γ) Enzyme-linked Immunospot (EliSpot) assay. In short, after PBMC isolation from whole blood samples using discontinuous density gradient centrifugation and resuspension in culture medium, which was comprised of RPMI1640 (Lonza, Vervies, Belgium, #BE12-702F) with 10% of human AB serum (C.C.pro, Oberdorla, Germany, #S-41-M), at a concentration of 1 x 10^7^ cells/ml, the cells were plated in 24-well plates for overnight resting. On the next day, the rested PBMCs were transferred to anti-IFN-γ pre-coated EliSpot plates (Lophius Biosciences, Regensburg, Germany, #12100010) in co-culture with specific antigens of interest for 16–18 h at a density of 2.5 x 10^5^ cells/well. Overlapping peptide pools against the following peptides were used for stimulation at a final concentration of 1 μg of each peptide/ml peptide pool: SARS-CoV-2 membrane protein (M, #130-126-703), nucleocapsid protein (N, #130-126-699), and CMV phosphoprotein 65 (pp65, #130-093-435) (all Miltenyi Biotec, Bergisch Gladbach, Germany) as well as SARS-CoV-2 proteins S1 and S2 (PM-WCPV-S), huCoV epitopes (strains 229E, #PM-229E-S-1 and OC43, #PM-OC43-S-1; S1 and S2), and antigens derived from human RSV (nucleoprotein, NP, #PM-HRSVB-NCPN) and IAV (matrix protein 1, MP1, PM-INFA-MP1-H1N1) (all JPT, Berlin, Germany). A positive control (PC) consisted of cells stimulated with staphylococcal enterotoxin B (1 μg/ml, SEB, Merck, Taufkirchen, Germany, #S4881) while PBMCs in culture medium alone served as a negative control (NC). The detection of IFN-γ was achieved using streptavidin-alkaline phosphatase (Mabtech Stockholm, Sweden, #12360002-S) and 5-13 bromo-4-chloro-3-indolyl phosphate/nitroblue tetrazolium (BCIP/NBT Liquid Substrate, SERVA, Heidelberg, Germany, #15246.01). Emerging IFN-γ spots were counted on an AID iSpot spectrum reader system using the AID EliSpot 8.0 Software (both from AID, Strassberg, Germany). For duplicate wells, means were calculated and for each specific antigen, the result was expressed as the number of spots per well (spw). For determining non-responders to this assay, a threshold was set at ≥3 spw or >2xNC. Additionally, spots/10,000 CD3^+^ T cells were calculated based on staining of PBMCs with anti-CD3 FITC (BD Biosciences, #345764), anti-CD4 PerCP (BD Biosciences, #345770), anti-CD8 APC (BD Biosciences, 345775), and anti-CD45 APC-H7 (BD BioSciences, #641417) followed by flow cytometric analysis.

### Intracellular Cytokine Staining in Antiviral T Cells

Intracellular cytokine staining for characterization of antiviral memory T-cell subsets was performed and analyzed as previously described (23). Briefly, 1 x 10^6^ isolated PBMCs were rested overnight in TexMACS culture medium (Miltenyi Biotec, #170-076-307) and then were stimulated with overlapping peptide pools derived from SARS-CoV-2 proteins M, N, S1 and S2, huCoV S proteins (strains 229E and OC43) as well as RSV NP, IAV MP1, and CMV pp65 (Miltenyi Biotec and JPT, catalog numbers see above) at a final concentration of 1 μg of each peptide/ml peptide pool. Stimulation with phorbol 12-myristate 13-acetate (PMA; 10 ng/ml, #P1585) and ionomycin (500 ng/ml, Sigma Aldrich, #I9657) as well as CytoStim (Miltenyi Biotec, #130-092-172) served as a positive control whereas unstimulated PBMCs were used as a negative control. After one hour of stimulation, 5 μg/ml of Brefeldin A (BioLegend, #420601) were added to each well, incubating for another 4 h afterwards. Following the total 5 h of stimulation period, the protocol included harvesting and extracellular staining with anti-CD4 PerCP (BioLegend, #, anti-CD8 PE-Cy7 (BioLegend, #344712), anti-CD45 Pacific Blue (BioLegend, #304022), anti-CD45RA BV605 (BioLegend, #304134), and anti-CD62L FITC (BioLegend, #304804). Thereafter, cells were fixed and permeabilized using the IntraPrep Kit according to the Manufacturer's instructions (Beckman Coulter, #A07803), followed by intracellular staining with anti-TNF-α (tumor necrosis factor alpha) APC (BioLegend, #502912) and anti-IFN-γ PE (BioLegend, #502509). Flow cytometric measurements were performed on a FACSCanto 10c system (BD Biosciences), acquiring at least 50,000 events in the CD45^+^ lymphocyte gate for each analysis.

### Data Analysis

The following software was used for data analysis: Microsoft Excel 2010 (Microsoft Corporation, Redmond), FlowJo™ v10 (FlowJoTM LLC, BD Biosciences), and BD FACSDiva v8.0.1 (BD Biosciences). Graphic visualization and statistical analysis were performed using Prism Version 8.2.0 (GraphPad Software, San Diego, California, USA), which also included Linear Regression and Kruskal-Wallis or Friedman Test followed by multiple comparison correction. Significance levels were calculated and expressed as *p*-values (^*^p < 0.05, ^**^p < 0.01, ^***^p < 0.001, ^****^p < 0.0001).

## Results

### Setting Up a Scale for Donation Times in Relation to Symptom Onset

**Figure 1:**
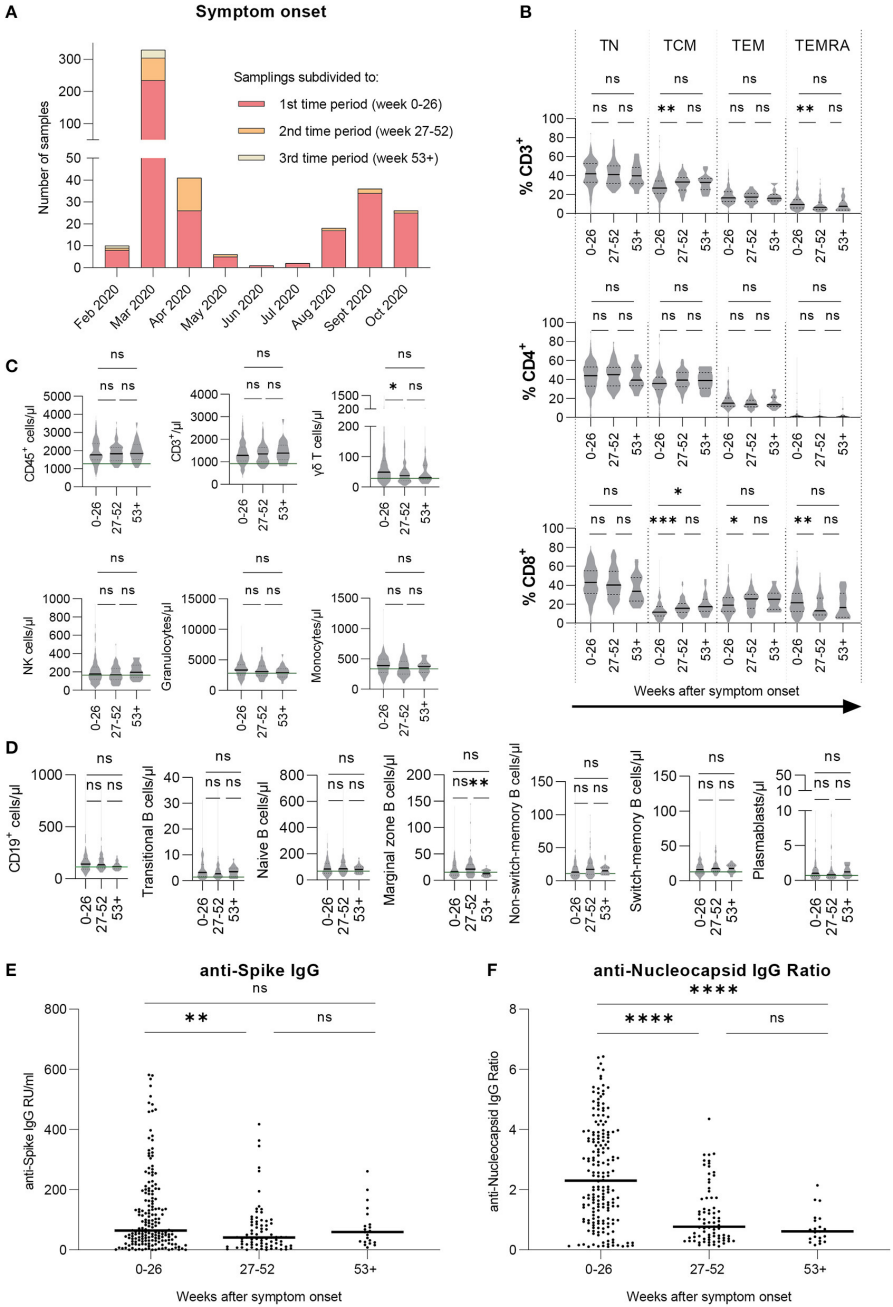
Cellular immune profile and T cell phenotypes are stable over time, SARS-CoV-2 specific antibody levels decrease and then remain stable over time. Immune cell counts and T cell phenotypes were assessed by flow cytometric analysis and serological ELISA assays were performed to measure antibody levels. Results are shown for our large cohort of COVID-19 recovered donors (total *n* = 206 samples). **(A)** Symptom onset of every donor's sample, subdivided into three time periods with respect to time after symptom onset. **(B)** T cell phenotypes for CD3^+^, CD4^+^, and CD8^+^ cells in percentages. **(C,D)** Cellular immune profile expressed as blood cell counts per microliter. **(E)** Levels of anti-SARS-CoV-2 Spike IgG antibodies expressed as RU/ml. **(F)** Levels of anti-SARS-CoV-2 Nucleocapsid IgG expressed as IgG ratios. **(B–F)** Values are presented over the course of three time periods: 0–26 weeks (*n* = 193), 27–52 weeks (*n* = 82), and 53+ weeks (*n* = 24) post symptom onset. **(B–F)** Kruskal-Wallis test followed by Dunn's multiple comparison. ^*^p < 0.05, ^**^p < 0.01, ^***^p < 0.001, ^****^p < 0.0001, ns = non-significant.

Sampling times were divided into three time periods depending on the time between symptom onset and sample collection: the first time period was defined as 0–26 weeks (*n* = 193 donors), the second time period was defined as 27–52 weeks (*n* = 82 donors), and lastly, the third time period was defined as 53 weeks and more (*n* = 24 donors) after symptom onset (Table 1). Figure 1A shows the distribution of sampling times in relation to time of symptom onset. The column's subdivisions reflect the three time periods described above. Notably, the distribution of symptom onset times is comparable to the general incidence of SARS-CoV-2 infections in Germany during that time (24). Most of the donors included in this study had a mild course of the disease (WHO clinical progression scale 2). The distribution of disease severity according to the time after symptom onset is evenly distributed amongst the three time periods. Due to the fact that from some donors, several samples were collected within one time period, we uniformly selected the first donation in each time period.

### Cellular Immune Profiling Reveals Mostly Consistent Blood Cell Counts Over Time

Whole blood samples were analyzed via flow cytometry to generate a cellular immune profile over the course of the study. The results are shown either as percentages or as cells per microliter blood (Figures 1B–D). CD4^+^ T cell phenotypes were stable while there were rises in CD8^+^ T central memory (TCM) cell counts as well as decreases in T effector memory re-expressing CD45RA cells (TEMRA) respectively (Figure 1B) from the first to the second time period. Additionally, CD8^+^ T effector memory (TEM) cells increased similarly (Figure 1B). Some innate and adaptive immune cell types, namely γδ T cells, granulocytes, and monocytes (Figure 1C) as well as CD19^+^ cells (Figure 1D), showed a slight progression toward median values that were observed for healthy, SARS-CoV-2-naïve donors in a previous study (23), indicating a longer retention time in the blood after the initial immune response. Interestingly, marginal zone B cell counts decreased significantly from the second to the third time period. To conclude, while there were minor changes in the CD8^+^ T cell compartment, most immune cell subsets remained stable over time.

### SARS-CoV-2 Specific Antibody Levels Mainly Decrease With Varying Consistency

Serum and plasma samples were analyzed for levels of anti-S and anti-Nucleocapsid protein (N or NCP) IgG antibodies. Results are shown as anti-Spike IgG RU/ml and IgG NCP Ratio. Median antibody levels against SARS-CoV-2 S (Figure 1E) as well as NCP (Figure 1F) significantly decreased toward the second time period and then remained on a stable level from the second to the third time period. Notably, highest measured values were consistently receding over time for S and NCP. In summary, levels of anti-S and anti-NCP IgG antibodies appeared to decrease toward a lower but stable level over time with anti-NCP IgG levels presenting an overall stronger decline.

### SARS-CoV-2 Specific T Cells are Detectable More Than a Year After Infection

**Figure 2:**
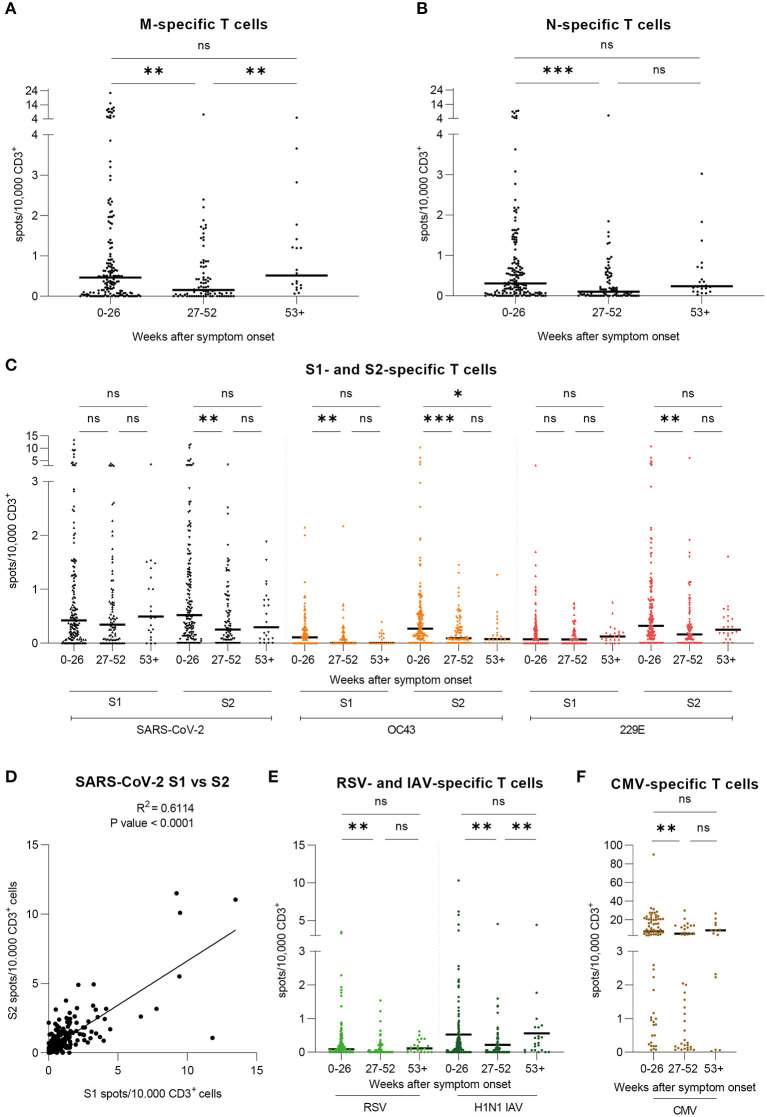
T cell frequencies against SARS-CoV-2, huCoV OC43 and 229E, RSV, IAV, and CMV epitopes are detectable and variously stable over time. Specific T cell frequencies were assessed by IFN-γ-EliSpot. Results are shown for our large cohort of COVID-19 recovered donors (total *n* = 206 samples) over the course of three time periods: 0–26 weeks (*n* = 193), 27–52 weeks (*n* = 82), and 53+ weeks (*n* = 24) post symptom onset. Values are expressed as spots per 10,000 CD3^+^ cells. **(A)** T cell frequencies against the peptide pool derived from SARS-CoV-2 Membrane glycoprotein (M). **(B)** T cell frequencies against the peptide pool derived from SARS-CoV-2 Nucleocapsid protein (N). **(C)** T cell frequencies against the peptide pools derived from SARS-CoV-2, huCoV OC43, and huCoV 229E Spike S1 and S2 subunit proteins **(D)** Correlation of T cell frequencies against SARS-CoV-2 Spike S1 and S2 subunits. R^2^ = 0.6114; *P*-value < 0.0001. **(E)** T cell frequencies against the peptide pools derived from RSV Nucleocapsid phosphoprotein (NP) and H1N1 IAV Matrix protein 1 (MP1). **(F)** T cell frequencies against the peptide pool derived from CMV phosphoprotein 65 (pp65). Results are shown for seropositive donors only (n=84) over the course of three time periods: 0-26 weeks (*n* = 67), 27–52 weeks (*n* = 28), and 53+ weeks (*n* = 12) post symptom onset. **(A–C,E,F)** Kruskal-Wallis test followed by Dunn's multiple comparison. ^*^p < 0.05, ^**^p < 0.01, ^***^p < 0.001, ^****^p < 0.0001, ns = non-significant.

Implementing IFN-γ EliSpot, we investigated T cell frequencies against overlapping peptide pools derived from SARS-CoV-2 and huCoV strains OC43 and 229E as well as RSV, IAV, and CMV. Results are shown as spots per 10,000 CD3^+^ T cells. We found SARS-CoV-2 M- and N-specific T cells to be stable on the long-term despite decreased frequencies in the second time period (Figures 2A,B). When evaluating T cell frequencies for SARS-CoV-2 S, S1 subunit-specific T cell frequencies appeared to be stable over the period of observation while S2 subunit-specific T cell frequencies decreased initially and then remained on a stable level toward the third time period (Figure 2C), similarly to SARS-CoV-2 N-specific T cells. Furthermore, we investigated the relation between the progression of T cell frequencies against SARS-CoV-2 and huCoV strains OC43 and 229E S1 and S2 subunits (Figure 2C). Within one virus strain, values for S2-specific T cells were generally higher than for S1, with generally highest values observed for SARS-CoV-2. Over time, T cell frequencies were stable for huCoV 229E S1 as well as for 229E S2 and OC43 S1, despite decreased levels in the second time period. The huCoV OC43 S2-specific T cell response was initially higher and then decreased to a stable level 6 months post symptom onset. By correlating the values for T cell frequencies against the S1 with the S2 subunit we found a strong level of correlation for SARS-CoV-2 (Figure 2D) and lower levels of correlation for the huCoV strains OC43 and 229E (data not shown). Lastly, we also analyzed T cell frequencies against RSV, IAV (Figure 2E), and CMV (Figure 2F). Lowest values were observed for RSV with a pattern similar to the one observed for SARS-CoV-2 S2. For IAV, T cell frequencies were comparable to those seen against SARS-CoV-2. Here, we saw a progression similar to that of SARS-CoV-2 M. Finally, largely stable T cell frequencies against CMV were detectable in CMV-seropositive donors. In summary, T cell responses against SARS-CoV-2 epitopes were detectable over the entire course of the study, with the most stable frequencies having been measured for SARS-CoV-2 S1 while values for SARS-CoV-2 S2 initially decreased and remained stable afterwards.

### Individual T Cell Frequencies Converge to Stable Level in Long-Term Follow Up

**Figure 3:**
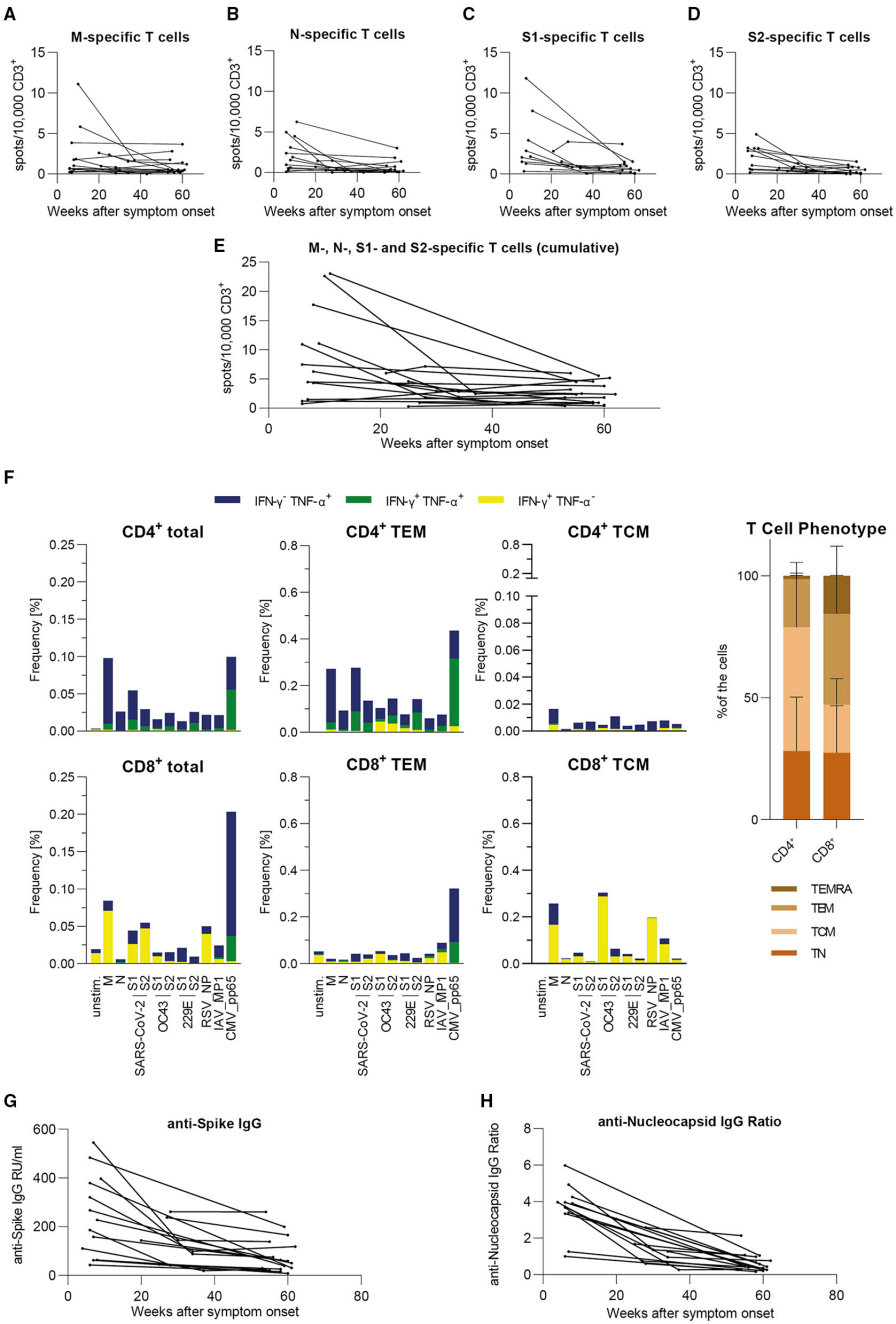
In our small individual cohort, T cell frequencies either are stable or decrease to a stable level over time, antibody level progressions vary from strong declines to stable levels over time. Specific T cell frequencies were assessed by IFN-γ-EliSpot, intracellular cytokines were detected by flow cytometric analysis and antibody levels were measured by serological ELISA assay. Results are shown for our small cohort of COVID-19 recovered donors (*n* = 18). **(A–D)** Individual T cell frequencies against peptide pools derived from SARS-CoV-2 M, N, S1, or S2. Only responding donors to each respective peptide pool are shown. **(E)** Cumulative individual T cell frequencies against peptide pools derived from SARS-CoV-2 M, N, S1, and S2. All donors in this cohort are shown. **(A–E)** Values are expressed as spots per 10,000 CD3^+^ cells. **(F)** Intracellular cytokine staining and FACS analysis of PBMCs after 5 h of stimulation with the indicated peptides. Results are presented as frequencies of IFN-γ^−^/TNF-α^+^ (blue), IFN-γ^+^/TNF-α^+^ (green), and IFN-γ^+^/TNF-α^−^ (yellow) among total T cells as well as T effector memory (TEM: CD45RA^−^CD62L^+^) cells and T central memory (TCM: CD45RA^−^CD62L^−^) cells within CD4^+^ and CD8^+^ T cell subsets [*n* = 8; CMV pp65: seropositive only (*n* = 6)]. Overall distribution of T cell phenotypes are displayed in the bar graph on the right side (mean + SD). **(G)** Individual levels of anti-SARS-CoV-2 Spike IgG antibodies expressed as RU/ml (RU = Relative Units). **(H)** Individual levels of anti-SARS-CoV-2 Nucleocapsid IgG antibodies expressed as IgG Ratios. **(A–E,G,H)** Results are divided into three time periods: 0–26 weeks (*n* = 18), 27–52 weeks (*n* = 7), and 53+ weeks (*n* = 18) post symptom onset.

We analyzed samples obtained from 18 donors individually in order to assess their individual progressions of SARS-CoV-2-specific T cell frequencies and antibody levels. This cohort consists of nine female and nine male donors, ranging from 24 to 68 years of age (Median = 49 years) and having shown mostly mild symptoms during infection [WHO score 2 (*n* = 17) and 4 (*n* = 1, hospitalized)] (Table 1). The results are expressed as spots per 10,000 CD3^+^ T cells with the T cell frequencies shown for SARS-CoV-2 M, N, S1, and S2 separately (Figures 3A–D) as well as their cumulative values (Figure 3E). For the analysis of each peptide pool, the respective non-responders to that peptide pool were excluded whereas in Figure 3E all donors are shown, since every donor responded to at least one peptide pool in each time period. We observed that donors with high frequencies in the beginning presented lower levels over the course of time. Individuals who showed equally low levels of T cell frequencies at the beginning presented consistent levels one year later. Interestingly, in two donors we detected a slight rise of SARS-CoV-2-specific T cell frequencies throughout time of convalescence. Furthermore, initial T cell frequencies were higher against SARS-CoV-2 M and S1 than against the other viral proteins. To characterize the specific long-term antiviral T cell populations (53–60 weeks post symptom onset) in more detail, we performed intracellular staining for IFN-γ and TNF-α after stimulation with the same peptide pools used in the EliSpot assays. Consistent with the findings described above, we were able to detect specific T cells against all SARS-CoV-2 derived peptide pools (Figure 3F). Furthermore, the results showed a pattern largely comparable to our previous work (23). There, highest specific T cell frequencies were detected within the subset of T effector memory (TEM) cells, as is the case here. Interestingly, CD4^+^ T cell responses against SARS-CoV-2 S shifted from SARS-CoV-2 S2 toward SARS-CoV-2 S1 subunits, which is in line with the results from the EliSpot assay (Figure 2C). In summary, individual T cell frequencies appeared to converge from variously high levels to a stable level over the course of 1 year after infection, which resembles our findings in the large cohort.

### Individual Antibody Levels are Consistently Detectable Over Time in Long-Term Follow Up

For the cohort described above, results of serological analyses are presented individually. Antibody levels against S (Figure 3G) and NCP (Figure 3H) were both initially declining toward the second time period. Subsequently, progressions vary from stable to mildly decreasing toward the third time period with apparently more consistent values for anti-S IgG. To conclude, we also observed a similar progression of this individual cohort's antibody levels in comparison with the large cohort.

### T Cells React to Alpha and Beta Variants Similarly as to Wild Type SARS-CoV-2

**Figure 4:**
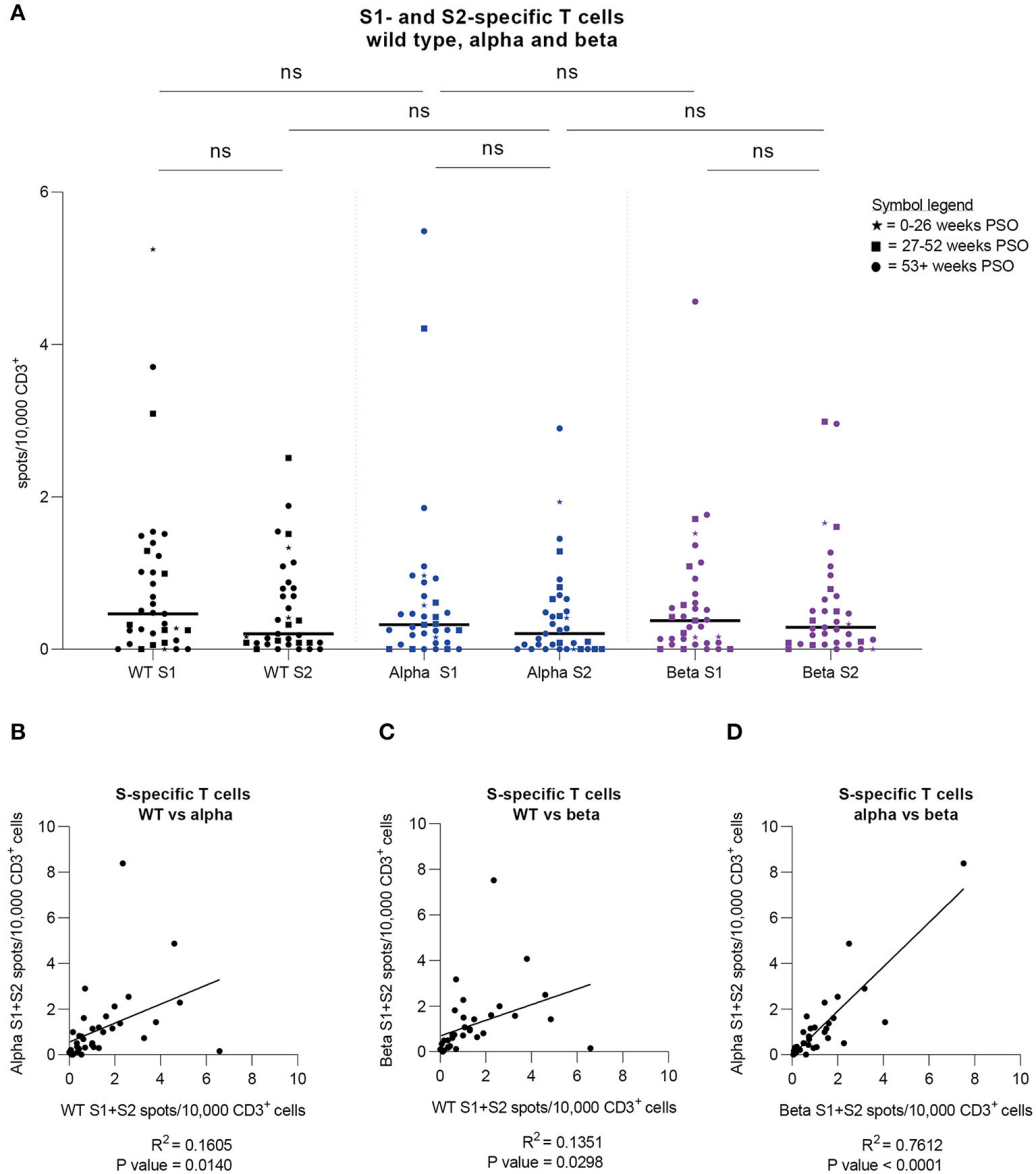
Strong cross-variant reactivity. Specific T cell frequencies and their correlations were assessed by IFN-γ-EliSpot against SARS-CoV-2 wild type as well as alpha and beta variants in a group of donors (*n* = 34). **(A)** T cell frequencies against peptide pools derived from SARS-CoV-2 Spike S1 and S2 subunits for wild type (WT) virus (black) as well as alpha (B.1.1.7; blue) and beta (B.1.351; purple) variants. Time periods of sampling are expressed with different symbols (⋆ = 0–26 weeks after symptom onset; ■ = 27–52 weeks after symptom onset; ● = 53+ weeks after symptom onset). Friedman test was followed by Dunn's multiple comparison and Wilcoxon matched-pairs signed rank test. ^*^p < 0.05, ^**^p < 0.01, ^***^p < 0.001, ^****^p < 0.0001, ns = non-significant. **(B)** Correlation of cumulative T cell frequencies against SARS-CoV-2 Spike S1 and S2 subunits for wild type virus (WT) and alpha variant. R^2^ = 0.1605; *P*-value = 0.014. **(C)** Correlation of cumulative T cell frequencies against SARS-CoV-2 Spike S1 and S2 subunits for wild type virus (WT) and beta variant. R^2^ = 0.1351; *P*-value = 0.0298. **(D)** Correlation of cumulative T cell frequencies against SARS-CoV-2 Spike S1 and S2 subunits for beta and alpha variant. R^2^ = 0.7612; *P*-value < 0.0001. **(A–D)** Results were detected by IFN-γ-EliSpot and are expressed as spots per 10,000 CD3^+^ cells.

Via IFN-γ EliSpot, we examined 37 samples from 34 donors for specific T cells against peptide pools derived from the S1 and S2 subunits of three SARS-CoV-2 variants, namely the wild type (previously presented), alpha (B.1.1.7), and beta (B.1.351) VOCs. This cohort consists of 14 females and 20 males, ranging from 25 to 69 years of age (Median = 46 years), who have shown mostly mild symptoms during infection [WHO score 1 (*n* = 1), 2 (*n* = 29), and 4 (*n* = 4, hospitalized)]. Samples were collected 21 to 61 weeks after symptom onset (Median 55.2 weeks) with the last sample collected in May 2021. Results are shown as spots per 10,000 CD3^+^ T cells. Considerably, no significant differences in T cell frequencies against each of the three variants were found (Figure 4A). When comparing S1 to S2, the T cell responses against the alpha and beta variants appeared to be lower for S2, as was the case for the wild type virus. We went on to correlate the cumulative T cell frequencies for each variant with each other. In this analysis, the alpha and beta variants showed the strongest correlation (R^2^ = 0.7612, *P*-value < 0.0001) while the correlations of alpha and beta variants to wild type SARS-CoV-2 both showed a similar but lower correlation level (alpha: R^2^ = 0.1605, *P*-value = 0.014; beta: R^2^ = 0.1351, *P*-value = 0.0298) (Figures 4B–D). Of note, all donors included in this study have most likely been infected with SARS-CoV-2 wild type virus due to the fact that the first case of a SARS-CoV-2 VOC infection in Germany occurred in late December of 2020 and the latest infection in this study was dated in October 2020 (25). In summary, T cell responses to S-derived peptide pools from wild type, alpha, and beta SARS-CoV-2 showed no significant differences and various degrees of correlation, of which the strongest correlation was observed between the alpha and beta variants.

### A Case Report of Reinfection With SARS-CoV-2

**Figure 5:**
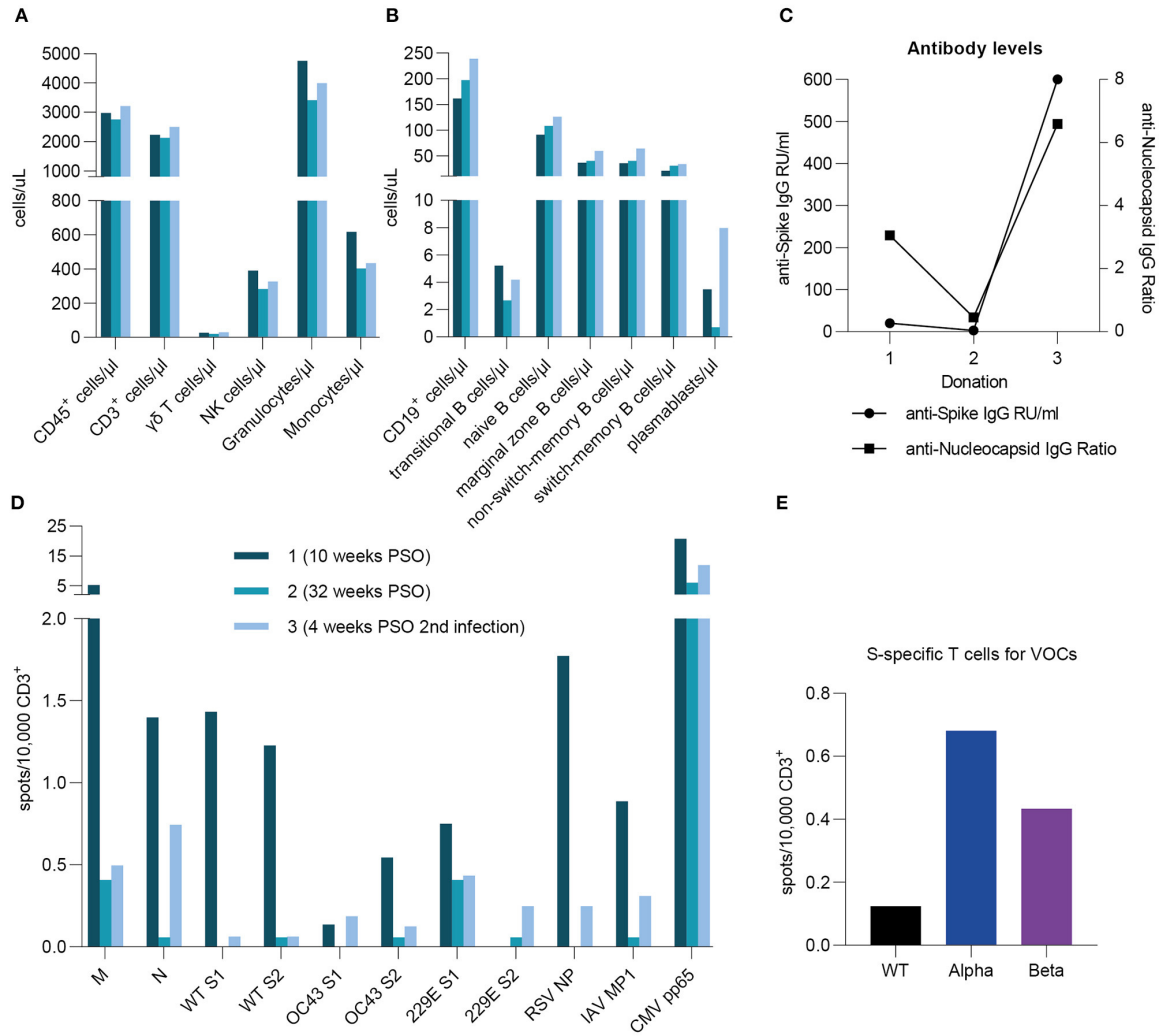
A case report of reinfection. A case reports presents specific T cell frequencies, immune cell counts, and antibody levels in one donor after primary infection (10 and 32 weeks post symptom onset) as well as after reinfection (4 weeks post symptom onset). The reinfection occurred 1 year after initial infection. **(A,B)** Immune cell counts were measured by flow cytometric analysis and are expressed as cells per microliter. **(C)** Anti-SARS-CoV-2 S- and NCP-specific antibody levels were assessed by serological ELISA assays and are shown as either IgG RU/ml or IgG Ratios. **(D)** Specific T cell frequencies against peptide pools derived from SARS-CoV-2, huCoV OC43, and huCoV 229E as well as RSV, H1N1 IAV, and CMV epitopes. **(E)** T cell frequencies against peptide pools derived from SARS-CoV-2 Spike S1 and S2 subunits for wild type virus (black) as well as alpha (B.1.1.7; blue) and beta (B.1.351; purple) variants. **(D,E)** Results were detected by IFN-γ-EliSpot and are expressed as spots per 10,000 CD3^+^ cells.

During the time of this study, one donor (male, >50 years old) became reinfected with SARS-CoV-2 almost 1 year after the first infection. Both infections were symptomatic with similar symptom durations. However, while during the first infection strong limb and internal pain was reported, symptoms during the second infection were milder (cough and elevated body temperature). We analyzed three samples from this donor, two of which were collected before the reinfection. Throughout this course of time, only small fluctuations for various immune cells were measured, indicating a rise directly after infection and a decrease over time (Figure 5A). We observed stable to slightly increasing counts of CD19^+^ cells, naïve, marginal zone, non-switched memory, and switched-memory B cells (Figure 5B). Notably, cell counts for transitional B cells and plasmablasts strongly decreased in the second sample, then rose again after reinfection with a more than two-fold increase in plasmablast levels compared to the first sample (Figure 5B). Strikingly, we saw a drastic increase in SARS-CoV-2-specific antibody levels, both against S and NCP, after reinfection (Figure 5C), which is consistent with the high cell count of plasmablasts. Moving on to the T cell frequencies (Figure 5D), it is remarkable that the majority of T cell responses after reinfection were lower than in the first donation. Interestingly, SARS-CoV-2 N-specific T cells appeared to be partially re-emerging after reinfection in this donor, whereas values for SARS-CoV-2 M resemble those of the second donation. Furthermore, frequencies for wild type SARS-CoV-2 S1 and S2 showed a strong decrease in the second donation, which is consistent in the third. Comparing those with the endemic coronaviruses, we also saw high initial T cell responses to OC43 S2 and 229E S1 decreasing to a stable level over time. On the contrary, OC43 S1 and 229E S2-specific T cells appeared more frequently after the reinfection with SARS-CoV-2. Lastly, in compliance with the general progression of T cell frequencies in this donor, RSV- and IAV-specific T cells were highest in the first sample, considerably lower in the second and increased again in the third, while T cell responses against CMV remained stable. Moreover, at the time of the third sampling, we compared the T cell responses to SARS-CoV-2 VOCs alpha and beta. As shown in Figure 5E, cumulative values for S1 and S2 were considerably low for the wild type virus and highest for SARS-CoV-2 alpha. Of note, the virus variant that caused the reinfection was not determined. In summary, in this case of reinfection with SARS-CoV-2, symptoms were milder but similar in duration. Re-exposure to SARS-CoV-2 was also followed by particularly high cell counts for plasmablasts and transitional B cells as well as distinctly increased antibody levels. The frequency of N-specific T cells increased upon reinfection, while it remained stable for the remaining tested antigens. Responses to VOCs were considerably higher than to wild type virus.

## Discussion

The collection of long-term data for cellular and humoral components of immune response and immunological memory in relation to SARS-CoV-2 is necessary to improve our understanding of the lasting immunity after overcoming a SARS-CoV-2 infection. With growing knowledge in this field, public health guidelines and vaccination protocols can be further improved to influence the dynamics of the COVID-19 pandemic. In this study, we analyzed antibody levels and antiviral T cell frequencies against structural proteins derived from SARS-CoV-2 and other respiratory viruses in mostly non-hospitalized patients with a wide range of symptoms over a period of more than one year post SARS-CoV-2 infection. Additionally, cellular immune profiling and T cell phenotyping were performed to assess the overall immunological status of the subjects in this study. We further present long-term analyses of individual follow-up samples in order to provide a more detailed view on the individual progression of cellular and humoral immunity toward SARS-CoV-2.

Our results reveal that in most cases, SARS-CoV-2 S-specific IgG levels remained stable over time and SARS-CoV-2 NCP-specific IgG levels decreased to a stable level within 6–12 months after symptom onset, while their range continuously diminished. Recent studies show that SARS-CoV-2-specific antibody levels after an infection were detectable in the majority of donors over a period of up to 1 year, even though the rate of recession varied (9, 11, 12, 26, 27). This supports our findings from the long-term analysis. Some studies show only similar progressions for anti-S and anti-N antibody levels over time (26, 27) whereas a study by Wang and colleagues assessed anti-RBD as well as anti-N antibodies and found stable levels of anti-RBD IgG compared to a reduction of anti-N IgG levels (11). This has also been observed in our results which suggest that the descent of anti-N IgG levels is more pronounced in comparison to the course of anti-S IgG. Additionally, Sandberg et al. present similar findings as they conclude that anti-S antibody levels appear to be more stable than those for anti-N (9).

Our data illustrate that SARS-CoV-2-specific T cells against SARS-CoV-2 epitopes were detectable over the entire course of the study, and showed most stable frequencies for SARS-CoV-2 S1 while values for SARS-CoV-2 S2 initially decreased and then remained stable. As already stated in previous research, SARS-CoV-2-specific T cells have been successfully detected for a period of up to 12 months post symptom onset (8–10, 12, 26). However, few studies have assessed T cell frequencies against SARS-CoV-2 M, N, and S separately over a period as long as 1 year. For shorter time periods, responses to SARS-CoV-2 proteins M, N, and S reveal comparable levels whereas other epitopes generally elicited weaker T cell responses (26, 28, 29). Interestingly, Lehman et al. scaled the antigen recognition of specific T cells against several SARS-CoV-2 derived peptides and found the recognition of SARS-CoV-2 M, N, and S derived peptides to be preferred over other SARS-CoV-2 epitopes with respect to individual variance for dominant epitope recognition (30). Notably, our analysis of individual T cell frequencies revealed similar findings in which every donor reacted to at least one SARS-CoV-2-derived peptide pool and the collective T cell responses of all donors were equally distributed among all investigated SARS-CoV-2 peptide pools. In addition, it was proposed that T cell responses positively correlate with the length of the respective peptides (30, 31), presenting another factor that is influencing the respective T cell response. However, the role of lasting cellular immunity that we observed still requires further investigation in regard to its ability to prevent or contain repeated infections with SARS-CoV-2 (32, 33). In this context, some COVID-19 patients presented with an overall impaired cellular immunity during primary infection (23). Thus, it is also of great importance to establish suitable therapies such as adoptive T cell transfer for cases of insufficient cellular immune response to SARS-CoV-2. In a recent phase 1 clinical trial by Pérez-Martínez and colleagues, the transfer of CD45RA-depleted memory T cell for treatment of hospitalized COVID-19 patients was shown to be feasible and safe (34).

We further examined and compared T cell responses to coronaviruses with other respiratory viruses such as RSV and IAV during the SARS-CoV-2 pandemic. While T cell responses against RSV remained stable, IAV-specific T cell frequencies slightly, yet not significantly, increased toward the third time period of the study. This increase could presumably be due to vaccination with certain IAV vaccines or contact with the virus, even though very few IAV infections were registered in Germany (35). In general, low T cell responses might correlate with fewer cases of infection with respiratory viruses during this pandemic, following the implementation of non-pharmaceutical interventions such as hygiene measures (36–38). These have shown to effectively reduce the transmission and thereby case numbers of SARS-CoV-2 (39–41). In this context, similar interventions were already successfully applied during the 2009 H1N1 influenza pandemic (42), which is in line with low incidences of other respiratory infections during this pandemic.

There is also great interest in the question of potential cellular and humoral cross-reactivity between immune responses against SARS-CoV-2 and endemic human coronaviruses. Some studies revealed that SARS-CoV-2 uninfected donors showed no or only low T cell responses to the viral proteins M and N while reacting more strongly to epitopes derived from SARS-CoV-2 non-structural proteins, possibly due to higher structural homology to other coronaviruses (7, 30, 43). This is in line with studies indicating higher correlations of immune responses between SARS-CoV-2, MERS-CoV, and SARS-CoV (31, 44). Mateus et al. (45) systematically selected SARS-CoV-2 epitopes based on this criterion and present similar findings while defining T memory cells as the reactive agents of cross-reactive immunological memory. Bacher et al. (46) observed higher cross-reactivity of SARS-CoV-2-specific memory T cells toward epitopes derived from common endemic coronavirus strains in contrast to memory T cells of common endemic coronavirus strains cross-reacting against SARS-CoV-2 Spike epitopes. Our results reveal generally higher T cell responses to SARS-CoV-2 in comparison to the endemic coronaviruses. Interestingly, we saw higher T cell responses against huCoV OC43 S2 and 229E S2 subunits but in contrast, SARS-CoV-2- specific T cells showed a higher response to S1 subunits over S2 subunits in the long-term. A higher response to SARS-CoV-2 S2 over S1 was observed for the humoral immune response (47) and attributed to the stronger homology of the S2 subunit to other coronaviruses (48). Subsequently, cross-reactive T cells were shown to be S2-specific (49), which is one possible explanation for our findings in this context.

Cross-reactivity is not only important regarding endemic coronaviruses but poses also a major concern in respect of emerging SARS-CoV-2 VOCs. Since the first case of a SARS-CoV-2 VOC infection occurred late in December 2020 in Germany and the latest infection in our cohort was dated in October 2020, it is of high likelihood that in the present study, all donors were infected with the SARS-CoV-2 wild type virus (25). The presented results therefore indicate a strong T cell cross-functionality toward SARS-CoV-2 virus variants alpha and beta. Some studies have found that SARS-CoV-2-specific antibodies from convalescent plasma donors appear to retain their neutralizing abilities against B.1.1.7 and lose them against B.1.351 (17, 19, 20). Based on known SARS-CoV-2-specific T cell epitopes and observed mutations within them, a similar progression has been proposed by Altmann et al. (50) for cellular immunity against upcoming virus variants. Some studies show slightly reduced T cell responses against virus variants after vaccination (51, 52), while Woldemeskel et al. found stable cross-variant T cell responses against SARS-CoV-2 (53). This finding is supported by Tarke et al. (33) who directly compared COVID-19 recovered as well as vaccinated individuals by implementing variant-specific overlapping peptide pools and observed no significant cross-variant differences in T cell responses. Interestingly, they synthesized their peptide pools based on known mutations in the viral genomic sequences of the respective VOC and thus propose that SARS-CoV-2 T cells keep their functionality across virus variants because the majority of epitopes is conserved (33). Our results are in compliance with these findings as they suggest generally equal T cell responses toward alpha and beta variants. This has to be further evaluated especially for recent variants such as the delta variant (B.1.617.2), as the number of reported infections after vaccination is rising (54, 55).

We present one case of reinfection with SARS-CoV-2, which was associated with a generally moderate elevation of antiviral T cell frequencies and a strong increase in the magnitude of the humoral response. So far, reinfections are rare as some studies show primary infections to generate a protective immune response in most cases (56–59). Nonetheless, various case reports describe reinfections often being associated with low immune responses during the first infection as well as different strains of SARS-CoV-2 infecting one individual (60–63). Although the individual in the present study presented clear signs of a specific immune response after the first infection, immune cells, antibody levels and SARS-CoV-2-specific T cells decreased within 32 weeks post symptom onset. However, the considerable increases of plasmablast counts and antibody levels as well as selected antiviral T cell frequencies after the second infection suggest a highly functional and rapid reaction upon second contact with the virus. Moreover, we suspect that the donor was reinfected with another virus variant due to the fact that after the second infection, T cell responses against VOCs alpha and beta where higher compared to those against wild type SARS-CoV-2, consequentially increasing the chance of reinfection. As such case report numbers are rising, so is the importance of defining parameters of insufficient immune responses in order to identify individuals with a higher risk of reinfection or infection after vaccination. This will be necessary to prevent infections in the future, for which many measures are possible depending on the individual's lasting immunity with boosting vaccinations as one potential option (64).

In conclusion, following convalescent COVID-19 patients for a period of more than 1 year after an infection with SARS-CoV-2, we detected T cell responses to SARS-CoV-2, huCoV strains OC43 and 229E, as well as RSV, IAV, and CMV over this whole time with varying stability for the different epitopes. Furthermore, we were able to measure antibody levels more than 1 year post infection. In the case of SARS-CoV-2 infection, values for humoral and cellular immunity mostly decreased and then remained at a stable level over time. Additionally, T cell reactivity toward SARS-CoV-2 VOCs alpha and beta was shown to be comparable to the T cell response against the wild type virus. Thus, our study provides evidence for long-term correlates of cellular and humoral immunity post SARS-CoV-2 infection as well as cross-variant T cell functionality. Based on these data, it will be necessary in the future to continue assessments of long-term immunity and to detect individuals with receding immunity in order to reduce the risks of reinfection, possibly by vaccination.

## Data Availability Statement

The original contributions presented in the study are included in the article/supplementary material, further inquiries can be directed to the corresponding authors.

## Ethics Statement

The studies involving human participants were reviewed and approved by Ethikkommission der Medizinischen Hochschule Hannover. The patients/participants provided their written informed consent to participate in this study. Written informed consent was obtained from the individual(s) for the publication of any potentially identifiable images or data included in this article.

## Author Contributions

AB, GB, RB, and BE-V: conceptualization. DG, AB, AC, MS, and ST-Z: methodology and validation. DG and AB: formal analysis and visualization. DG, AB, AC, PM, ST-Z, MS, and NG: investigation. UK, GB, RB, and BE-V: resources. DG, AB, PM, and BE-V: writing and original draft. AB, ST-Z, and BE-V: supervision. AB and BE-V: project administration. RB and BE-V: funding acquisition. All authors contributed to the article and approved the submitted manuscript.

## Funding

This study was funded in part by the German Research Foundation (DFG; Research Unit 2830), the State of Lower Saxony (14-76103-184 CORONA-12/20) and the Federal Ministry of Health (ZMVI1-2520COR804).

## Conflict of Interest

UK is employed by the Helmholtz Centre for Infection Research. The remaining authors declare that the research was conducted in the absence of any commercial or financial relationships that could be construed as a potential conflict of interest.

## Publisher's Note

All claims expressed in this article are solely those of the authors and do not necessarily represent those of their affiliated organizations, or those of the publisher, the editors and the reviewers. Any product that may be evaluated in this article, or claim that may be made by its manufacturer, is not guaranteed or endorsed by the publisher.
